# Evaluation of Biomechanical and Chemical Properties of Gamma-Irradiated Polycaprolactone Microfilaments for Musculoskeletal Tissue Engineering Applications

**DOI:** 10.1155/2022/5266349

**Published:** 2022-04-29

**Authors:** Laura Rojas-Rojas, Andrea Ulloa-Fernández, Silvia Castro-Piedra, Walter Vargas-Segura, Teodolito Guillén-Girón

**Affiliations:** ^1^Material Science School, Technological Institute of Costa Rica, Cartago, Costa Rica; ^2^Physics School, Technological Institute of Costa Rica, Cartago, Costa Rica; ^3^Biology School, Technological Institute of Costa Rica, Cartago, Costa Rica; ^4^Radiotheraphy Department, Hospital Mexico, San José, Costa Rica

## Abstract

An appropriate and reliable sterilization technique is crucial for tissue engineering scaffolds. Skeletal muscle scaffolds are often fabricated using microfilaments of a wide variety of polymers. One method for sterilization is 25 kGy of gamma irradiation. In addition, sterilization through irradiation should administer a dose within a specific range. Radiation directly affects the chemical and mechanical properties of scaffolds. The accuracy and effects of irradiation are often not considered during sterilization procedures; however, these are important since they provide insight on whether the sterilization procedure is reliable and reproducible. This study focused on the chemical and mechanical characterization of 25 kGy gamma-irradiated scaffold. The accuracy and uncertainty of the irradiation procedure were also obtained. X-ray diffraction (XRD) and differential scanning calorimetry (DSC) analyses were performed to determine whether the crystallinity of the polymer changed after irradiation and whether gamma rays influenced its thermal properties. The tensile parameters of the microfilaments were analyzed by comparing irradiated and nonirradiated scaffolds to determine whether gamma radiation changed their elastic behavior. Dose distribution and uncertainty were recorded with several dosimeters. The results showed that the irradiation process slightly affected the mechanical parameters of the scaffold; however, it did not modify its crystallinity or thermal properties. The irradiation was uniform, since the measured uncertainty was low. The scaffold was pathogen-free after 7 days; this meant sterilization was achieved. These results indicated that gamma-sterilized scaffolds were a promising material for use as a skeletal muscle analog material for tissue-engineering applications because they can be sterilized with gamma rays without changing their chemical structure and mechanical properties. This study provided the dose distribution measurement and uncertainty calculations for the sterilization procedure.

## 1. Introduction

Biocompatible polymers are frequently used in medical and tissue-engineering applications because of their properties and the wide variety of fabrication techniques available to produce different and complex geometries, patterns, and structures [1–3]. Filaments with diverse diameters and configurations are often used in muscular tissue-engineering applications [4, 5]. These structures can be fabricated using PCL, which is a semicrystalline polymer with a low melting point, and it is biocompatible and biodegradable. Several PCL structures are fabricated by electrospinning, and this produces interlaced or parallel fibers, mostly in a 2D configuration with diameters that vary from nanometers to millimeters [6, 7]. For 3D configurations, other fabrication approaches have to be used: long, individual PCL microfibers can be fabricated by extrusion with a small extrusion dye using a procedure similar to that reported by An et al. [8]. A long, uniform microfilament can be fabricated and organized to mimic skeletal muscle configuration. Further, microfilaments and fibers can be used in prosthetics and other fields [9].

An adequate scaffold for muscular tissue engineering should allow for cell adhesion and provide a suitable microenvironment for cell proliferation and migration [10]. Several cell types can be used for skeletal tissue engineering [11]. Muscle tissue engineering often uses C2C12 CRL-1772^TM^ (ATCC) cells because these are myoblast cell lines that can be differentiated, expressing muscle proteins; in combination with an appropriate scaffold, it can provide a suitable model of skeletal muscular constructs [12]. The applications of cell-seeded scaffolds include basic biological studies, drug analysis, or disease models [13, 14]. A scaffold must be sterile for tissue-engineering applications. Sterilization is a process in which a sample is made free of pathogens or biological organisms such as fungi, bacteria, viruses, or spores [15]. Sterilization methods include heat treatment, irradiation, plasma, chemical sterilization, and other novel techniques [16–18]. Recently, D'Amico et al. used microwaves at a power of 1800 W and 85°C for instrument sterilization [19]. Each sterilization method has different operating conditions such as energy, temperature, and exposure time; this can influence the scaffold morphology, structure, and mechanical resistance to deformation and degradation.

Gamma radiation is an ionizing sterilization process in which a sample is exposed to gamma rays to eliminate microorganisms that can be present on the sample [20]. Highly energetic gamma rays interact with matter principally by ionizing molecules; for example, water molecules undergo hydrolysis, which produces hydroxyls [21]. The hydroxyls break down DNA present in pathogens and microorganisms and have a strong oxidant effect on other biological compounds [21]. The radiation dose was based on the initial microbial load and its degradation rate [20]. According to ISO 11137, the most common dose for irradiation sterilization is 25 kGy [20, 22]. The dose rate, applied dose, and time affect the internal structure of the irradiated sample in various ways because irradiation affects the chemical and physical structures of the materials [20]. Radiation can degrade the properties of the polymer by a process called scission; this involves breaking internal chemical bonds. If scission occurs, the mechanical properties of the polymer are weakened or diminished, which compromises its use in scaffolds. Another possibility attributed to ionizing radiation is cross-linking, and this produces more chemical bonds within molecules and can result in an increase in tensile strength [23, 24]. Gamma radiation has many advantages such that the sterilization method has a high penetration range and does not leave residues, and the temperatures remain mild [17, 18].

It is necessary to study chemical and mechanical properties of polymers because these properties may change because of matter-radiation interactions. The physical and chemical properties of irradiated polymers were characterized by infrared spectroscopy (FTIR), XRD, and DSC [25]. It is important to study whether a standard sterilization dose of 25 kGy modifies the mechanical properties of PCL microfilaments to confirm that the scaffolds are suitable for biomechanical and in vitro applications [26]. This study aims to investigate the effects of gamma ray sterilization on the mechanical properties of PCL-extruded microfilaments with the goal of providing the irradiation dose uncertainty assessment and whether the sterilized scaffold retained its properties.

## 2. Materials and Methods

### 2.1. Microfilament Preparation

PCL Mn 80000 (Sigma-Aldrich, Burlington, USA) pellets were used as the raw material to produce microfilaments. The microfilaments were fabricated using an extruder equipped with a die of 1 mm internal diameter and a tunable spooler. After cooling, the extruded filament was stretched until plastic yielding occurred, which produces a microfilament with an average diameter of 90.00 ± 3.85 *μ*m. It was stored in a controlled environment at 23°C and 40% humidity for the tests. Microfilaments were prepared for microbiological assays, DSC, or mechanical testing. XRD analysis was made to flat circular samples prepared by melting PCL at 60°C.  Table 1 summarizes the samples and tests conducted.

### 2.2. Irradiation of Samples

An Ob-Servo Ingis with a Co-60 source gamma irradiator (Izotop, Hungary) was used to sterilize the samples. It has 24 cobalt-60 pencil sources and a 15 cm diameter and 27 cm height sample chamber. The temperature inside the irradiation chamber was 31°C. Microfilaments and samples were placed inside a sealed bag for irradiation.

#### 2.2.1. Dose Mapping Procedure

**Figure 1:**
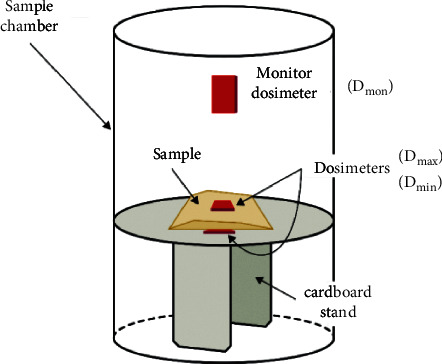
Sample and dosimeters inside the irradiation chamber of the Ob Servo Ignis.

A bag with the microfilaments was placed on the top of a cardboard stand located in the middle of the sample chamber shown in Figure 1. Two dosimeters were placed on the filaments: one over the bag and the other under the bag poly(methyl methacrylate) (PMMA); Perspex and Amber dosimeters (Harwell Dosimeters, UK) were used for minimum (*D*_min_) and maximum dose (*D*_max_) estimations. A monitoring dosimeter (*D*_mon_) was placed 3 cm above the sample holder. The dosimeter placement and measurements were repeated twice according to ISO 11137-3 [27]. *D*_min_, *D*_max_, and *D*_mon_ were calculated as the average of these three measurements.

ISO-11137-4 (2020) [28] was used to determine the overall uncertainty in the irradiation process (*σ*_proc_). It can be estimated by

(1) $$\begin{array}{c} \sigma_{\text{proc}}=\sqrt{\sigma_{\text{cal}}^{2}+\sigma_{\text{rep}}^{2}+\sigma_{\text{mach}}^{2}+\sigma_{\text{map}}^{2}},\; \end{array}$$

where *σ*_cal_, *σ*_rep_, *σ*_mach_, and *σ*_map_ represents the dosimeter calibration uncertainty, dosimeter reproducibility uncertainty, machine variability, and dose-mapping uncertainty, respectively. The guidelines were followed to calculate *σ*_cal_, as described by Sharpe and Miller [29]. The dosimeter reproducibility uncertainty (*σ*_rep_) was estimated by repeating the process; the monitoring dosimeter (*D*_mon_) was used to estimate its variation. The dose mapping uncertainty (*σ*_map_) was determined by repeating the mapping process, and it had two components: one for the minimum dose (*σ*_map,min_) and the other for the maximum dose (*σ*_map,max_).

*σ*_mach_ is related to the radiation source and conveyor system; four dosimeters were placed in the same location as the monitoring dosimeters in a circular configuration of 90° between them to determine this uncertainty. The machine variability (*σ*_mach_) incorporates variations in the position of the sample chamber inside the irradiator. Information on the uncertainties is summarized in Table 2 and organized according to the GUM [30].

Type A uncertainties were calculated by

(2) $$\begin{array}{c} \sigma \left(\%\right)=\frac{s_{D}}{\bar{D}\sqrt{n}}100, \end{array}$$

where s_D_, $\bar{D}$, and n represent the standard deviation of the dosimeter readings, average dose of the dosimeters, and number of measurements, respectively.

The dose range was determined as the dose range that guarantees the acceptance of the irradiated sample, D_ster_ is the desired sterilization dose for the sample, and D_max,acc_ denotes the maximum accepted dose. The dose range was calculated according to ISO 11137-4 [27] using

(3a) $$\begin{array}{c} D_{\text{target,lower}}=\frac{D_{ster}}{R_{\min/\text{mon}}}\frac{1}{1-k\left(\sigma_{\text{proc,min}}/100\right)}, \end{array}$$

(3b) $$\begin{array}{c} D_{\text{target,upper}}=\frac{D_{\text{max,acc}}}{R_{\text{max}/\text{mon}}}\frac{1}{1+k\left(\sigma_{\text{proc,max}}/100\right)}. \end{array}$$

where *R*_min/*mon*_ and *R*_*max*/*mon*_ denote the ratio of the maximum dose D_max_ and D_mon_ and ratio of the minimum dose D_min_ and D_mon_, respectively, in both cases determined by dose mapping. The coverage factor (k) was equal to 2 for a 98% confidence level, which considers a single-sided distribution function.

### 2.3. Microbiological Load Tests

#### 2.3.1. Reagents

For microbial testing, dehydrated thioglycollate broth medium (TB; Oxoid; Hampshire-UK), potato dextrose agar (PDA; Oxoid; Hampshire, UK), plate count agar (PCA; Oxoid; Hampshire, UK), and peptone water (Prelab, San Jose, Costa Rica); culture media were prepared following the manufacturer's instructions. These reagents were dissolved in distilled water and sterilized using steam at a temperature of 121°C for 15 min prior to use. Phosphate-buffered saline (PBS, Sigma-Aldrich, St. Louis, MO, USA) was used for cleaning or washing. Cell growth assays were performed with Dulbecco's Modified Eagle Medium (DMEM, Gibco, Paisley-UK) supplemented with 1% glutamine (GIBCO^TM^, Grand Island USA), 1% penicillin-streptomycin (GIBCO^TM^, Auckland-NZ), and 10% fetal bovine serum (GIBCO^TM^, Grand Island USA) as a growth medium for the C2C12 (CRL-1772^TM^) cell line. 3-(4,5-Dimethylthiazolyl-2)-2,5-diphenyl tetrazolium bromide (MTT MP pharmaceuticals; Illkirch, France) was used to measure cellular metabolic activity. The reagents used in this study were of analytical grade, and all organism proliferation methods were assessed using aseptic techniques and sterile conditions.

#### 2.3.2. Microbial Assay

The ten samples of the PCL 25 kGy group were washed with sterile PBS for 1 min and an initial estimation of the microbial load on the microfilaments was performed using the ISO 11137 standard [20]. A swap soaked in peptone water was rubbed onto the PCL 25 kGy samples to detect aerobic mesophilic microorganisms (AMM), yeast, and fungi (Y/F). Then, streaking on PCA and PDA was conducted, and the plates were incubated for 7 days at 37°C and 25°C, respectively. For aerobic (A), anaerobic (AN), and facultative (F) microorganisms, a swap soaked in peptone water was rubbed on the filaments and then rinsed in 5 ml TB tubes. The tubes were covered with sterile cotton and incubated for 7 days at 37°C. Seven days were selected for microorganism counting according to ISO 11737-1:2012 [31]. The same procedure for microbial load estimation was repeated after gamma irradiation.

### 2.4. Cell Growth on Microfilament Scaffold Study

Mouse myoblasts C2C12 (CRL-1772^TM^) were incubated on PCL microfilaments to evaluate the viability, adhesion, and proliferation of cells on the scaffold. Cells (5 × 10^4^) were seeded onto the scaffolds with the growth medium. The samples with cells were incubated under standard conditions (37°C and 5% CO_2_) for seven days. The microfilaments were monitored on days 3, 5, and 7. Three scaffolds were moved to a new empty well and incubated for 2 h with a solution of 10% MTT dissolved in fresh DMEM; the final concentration of MTT was 0.5 mg/ml. Control C2C12 cells were incubated using the same procedure but without microfilaments. Then, the medium was removed, and the produced formazan salts were diluted with 100% ethanol; the absorbance was measured using a plate reader FLUOStar Optima (BMG LABTECH) at 570 nm. The absorbance was analyzed for the microfilament scaffolds and control sample. This experiment was repeated twice, and the statistical differences between the number of cells were calculated for the monitored days (3, 5, and 7) using the Minitab 18 software (State College, PA, Minitab, Inc.)

### 2.5. Chemical Evaluation of Samples

Irradiated and nonirradiated PCL samples were studied using an XRD Panalytical Empyrean instrument. The XRD analysis was conducted with a copper tube (*λ* = 1.54 Å), 45 kV, 40 mA, and scanning range from 15° to 40°. A soller slit of 0.04 rad located at the X-ray tube and a large soller slit of 0.04 rad located at the detector were used. A divergence slit of 1/4° and antiscatter slit of 1/2° were also utilized. Further, K*β* was filtered using Ni. The software Data Collector, High Score Plus, and PDF4+ (2021) were used.

DSC analysis was performed using Instruments Discovery DSC 250 under nitrogen flow. Samples were cooled from 30°C to −80°C at a 10°C/min rate; then, there was a first heating from −80°C to 130°C at a rate of 10°C/min, cooling up to −80°C, and a second heating to 130°C at a rate of 10°C/min. The melting point was considered the maximum endothermic transition, and the enthalpy considered the area of the peaks during the heating cycle.

### 2.6. Tensile Testing

**Figure 2:**
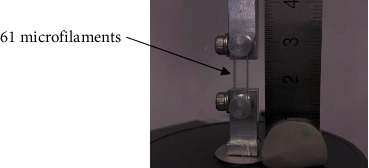
Filament arrangement in grip system used for tensile tests.

Monotonic tensile tests were performed on the PCL 25 kG and PCL 0 kGy samples. Each sample was composed of 61 filaments arranged on a grip system designed and fabricated for this purpose (Figure 2). The tensile test speed was set to 38 mm/min using USP NF24 [32]; it was elongated to 40% strain. The stress was calculated as the load divided by the equivalent area of the 61 tied filaments using the average diameter. The strain is the ratio of the displacement change to the initial microfilament length (l = 19 mm). The linear section from 8% to 15% strain of the stress behavior was used to obtain the Young's modulus (E). The yield tensile stress (*σ*_y_) was obtained as a 0.2% offset of the strain; the ultimate tensile stress (*σ*_ult_) was the maximum stress value registered during the test. Mean values and standard deviations are reported in this study. One-way ANOVA with Tukey pairwise comparisons was employed to determine statistically significant differences between mechanical parameters. The Minitab 18 software was used for the statistical calculations.

## 3. Results and Discussion

### 3.1. Irradiation of Samples

Dosimetry control and uncertainty studies were performed to guarantee the reproducibility of the irradiation processes and study the accuracy of the dose. The uncertainty budget for the irradiation components is listed in Table 3.

From the uncertainty of the components, the total uncertainty of the process was estimated as *σ*_proc,min_  = 3.18% and *σ*_proc,max_  = 3.65% for k = 1. In addition, *σ*_proc,min_  = 6.37% and *σ*_proc,max_ was 7.30% using a coverage factor of k = 2. The uniformity coefficient (*R*_max/min_) indicates the ratio of the maximum and minimum absorbed doses in the irradiated sample; the obtained result was 1.0038, which indicates a difference of 0.38% between the maximum and minimum doses. The closer *R*_max/min_ is to 1, the more uniformly distributed dose the sample receives; therefore, our result confirmed that the microfilament dose is uniform, which increases the confidence in the reading. Augustine [23] used 35 kGy for the sterilization of PCL. Based on this information and in accordance with ISO 11137-3 [28], a dose of 35 kGy was selected as D_max,acc_ in the microfilament irradiation process; D_ster_ was 25 kGy according to ISO 11737-2:2009 [31]. Finally, the limit values of the acceptance dose range for the monitoring dosimeter were calculated for routine dosimetry based on the uncertainties of the process. These values were calculated using equation (3a) and (3b).  Table 4 presents the results.

The positions of *D*_target,lower_ and *D*_target,upper_ allow the evaluation of the homogeneity and dose distribution on the irradiated material. Inside the irradiation chamber, the dose rate was not the same throughout its volume because of the variation in the activity of the Co sources. In all scenarios, it is critical to optimize the sterilization method to balance the level of sterility assurance without negatively affecting the product [33].

Sterilization using gamma rays is suitable for PCL microfilaments. Ob-Servo Ignis has a temperature of 31°C inside the sample chamber, which is sufficiently low to preserve the integrity of the microfilaments, and this is a great advantage of this sterilization process. In contrast, high temperature can modify the configuration of the microfilaments, which results in the modification of the 3D structure, alteration in the uniformity of the filament, or diameter variation. In addition, the selected sterilization process has several other advantages such as its high penetration range and energy, which allows packed samples to be sterilized. Further, sterilization occurs at atmospheric pressure and air atmosphere, without the use of toxic gases. Moreover, the dose can be confirmed and validated by dosimeter placement [34]. Other techniques such as ethanol and ultraviolet (UV) or ethylene oxide (EtOH) are commonly used for the sterilization of PCL [35]; however, their vapors and products are highly toxic.

### 3.2. Microbial Tests

The microbial load on the microfilaments was estimated to study the effectiveness of sterilization. These results are summarized in Table 5. Prior to sterilization, the microfilaments exhibited microbial loads comprising AMM, AN, A, F, Y, and F microorganisms.

**Figure 3:**
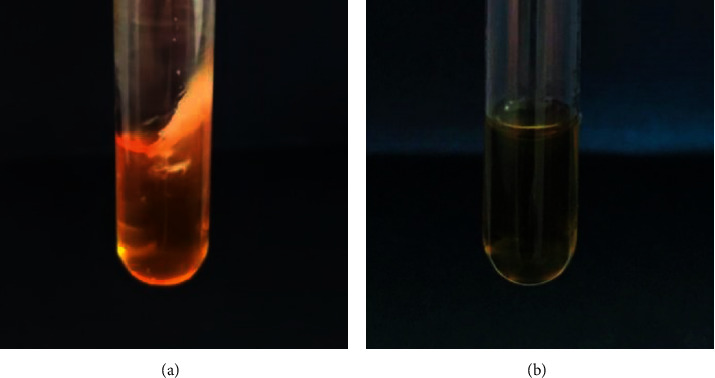
5 ml TB tubes in (a) without sterilization and in (b) after sterilization with 25 kGy of gamma rays.

After sterilization, there was a significant reduction in the number of microorganisms present in the microfilaments; AMM, A, AN, and F are eliminated. Samples 1–10 complied with the sterility test because no pathogens were detected after sterilization. An example of pathogen reduction is shown in Figure 3. Before sterilization, TB showed contamination as clouding of the culture broth; after sterilization, the TB tube became transparent (Figure 3(b)). These results showed that the sterilization method was useful for reducing the microorganisms in the microfilaments.

### 3.3. Cell Growth on Microfilament Scaffold Study

**Figure 4:**
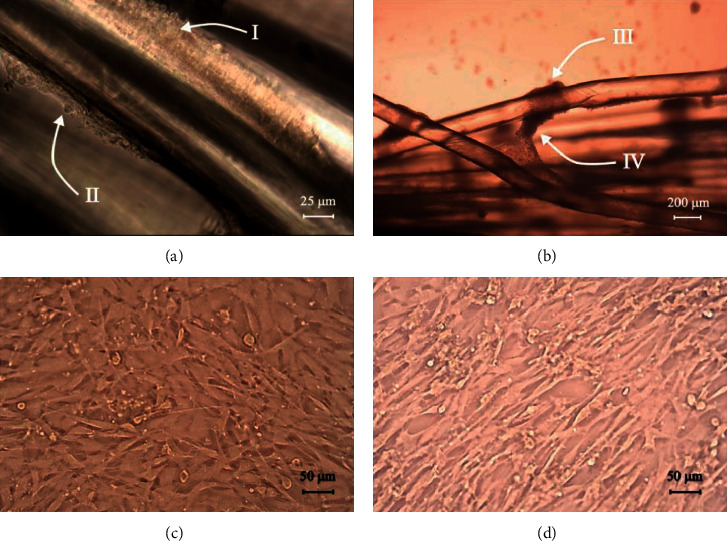
C2C12 growth at day 3 (a) and day 7 (b) on the microfilaments; the C2C12 control cells without scaffolds at days 3 (c) and 5 (d).

The C2C12 cells proliferated and grew on the PCL scaffolds. Figures 4(a) and 4(b) show myoblasts growing on the microfilaments. The white arrows depicted as I and II in Figure 4(a) display cells attached to the surface of the microfilaments at day three; the white arrows depicted as III and IV in Figure 4(b) show cell growth at day seven. Figure 4(b) presents evidence that after seven days in culture, the cells grew between the two filaments, and they were able to surround some sections of the microfilament. The PLC samples provided a suitable surface for cell attachment and proliferation.

The cells had a round morphology and clustered or gathered in certain places of the microfilaments; however, the cells did not show alignment along the scaffolds. Figures 4(c) and 4(d) show the control test as a monolayer of cells in a two-dimensional environment. The cells showed spindle-shaped myoblasts on days three and five. No mechanical tension was applied to the filaments during the cell culture test; therefore, no alignment or differentiation was expected on these types of cells [36]. However, it is important to consider for future work to apply the load to the filaments during cell growth to understand the behavior of these cells on filaments that could replace muscular tissue.

**Figure 5:**
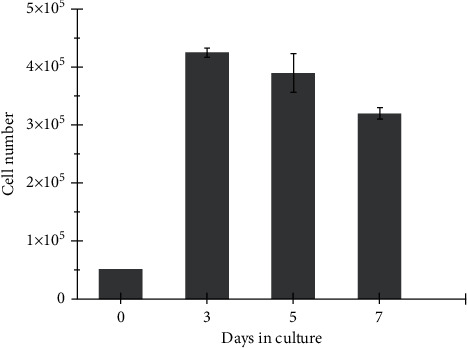
Viable C2C12 cells grown on the microfilament scaffold.

The cell proliferation was measured by the MTT test.  Figure 5 shows a significant increase in cell proliferation from day zero to day three. The cells proliferated from 5 × 10^4^% to 42 × 10^4^% after three days in culture. Next, cells slightly decreased from 42 × 10^4^% to 33 × 10^4^% from day three to day seven. Although the viable cell number was reduced, a statistical analysis indicated that there was no significant difference between the number of viable cells at days 3, 5, and 7 (p = 0.11 one-way ANOVA).

The main requirement for using biomaterials for tissue engineering is the acceptability and nontoxicity of the scaffold to cells and tissues. The results from the MTT assay showed metabolic activity for seven consecutive days; see Figure 5. Therefore, the scaffold was nontoxic. Further, the micrographs in Figure 4 show that the cells had the appropriate morphology and are distributed on the microfilament; thus, the scaffold is a favorable surface for cell growth. These results are related to those of Browe and Freeman [37], which showed that PCL is a biocompatible and biodegradable polymer.

### 3.4. Chemical Evaluation of the Irradiated Structure

**Figure 6:**
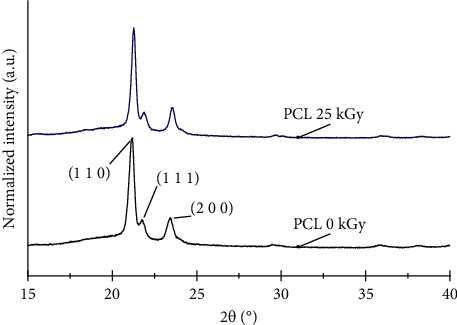
Diffractograms of (a) nonirradiated PCL and (b) irradiated PCL.

The XRD results indicated that PLC maintained its crystalline structure after gamma irradiation at 25 kGy.  Figure 6 shows that no new diffraction planes were present; no new symmetries were created by the irradiation process. Therefore, the crystalline structure of the PCL microfilaments remained the same. In addition, no translation of the peaks occurred, and there was no abrupt change in the intensity of the signals. The main reflection angles for irradiated and nonirradiated PCL were located at 21.2°, 21.7°, and 23.4°. These results are consistent with those of Paula et al. [38].

**Figure 7:**
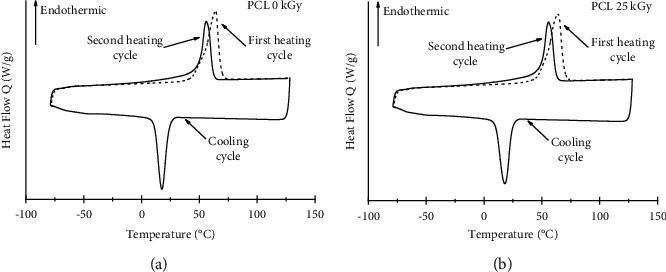
DSC curves of (a) 0 kGy PCL and (b) 25 kGy PCL.

The DSC analysis was used to study changes in the thermal behavior or modification of the crystallinity of PCL caused by sterilization.  Figure 7 depicts the resulting DSC thermal behavior for (a) PCL 0 kGy and (b) PCL 25 kGy. For PCL 0 kGy, the first heating process up to 130°C showed a melting peak at 64°C, with a melting enthalpy of 73 J/g. Then, the cooling cycle shows a phase transition at 17°C. The second heating step showed a melting peak at 56°C with an enthalpy of 39 J/g. The same behavior was observed for 25 kGy PCL as the melting temperature was 64°C for the first heating and 55°C for the second heating, with a phase transition at 17°C for the cooling cycle. Calculated enthalpies were 68 J/g and 43 J/g, respectively. Both samples exhibited the same DSC curve behavior and temperature. Microfilaments fabricated in this work are similar to those reported by Bosworth et al. [35], who reported a melting point of 56.76°C for the electrospun 25 kGy-irradiated PCL.

The irradiated and nonirradiated polymers retained their crystalline structures during both heating processes, with only a slight decrease in the melting point and enthalpy. This change was attributed to the polymer itself rather than the irradiation process because it was present in both irradiated and nonirradiated polymers. These results are in accordance with those of Navarro et al. [25], who reported that the crystallinity of PCL can be affected by gamma doses greater than 35 kGy.

### 3.5. Tensile Properties of the Microfilaments

The mechanical parameters of the PCL microfilaments are listed in Table 6. There was a slight reduction in *σ*_y_ and *σ*_ult_ after the samples were sterilized. The same behavior was observed for the elastic moduli of the microfilaments, and this decreased when the samples were sterilized with gamma rays. Changes in the tensile properties of PCL are associated with changes in its crystalline structure. The DSC and XRD results confirmed that the crystalline structure of the polymer remained the same after irradiation, and this was complementary to the variation in the mechanical parameters of the microfilaments.

One-way ANOVA statistical analysis of the mean E, *σ*_y_, and *σ*_ult_ values yielded p = 0.104, 0.064, and 0.248, respectively, which indicates that there were no statistical differences between the irradiated and nonirradiated microfilaments. The Tukey pairwise comparisons yielded that the means between groups were not statistically different from each other.

**Figure 8:**
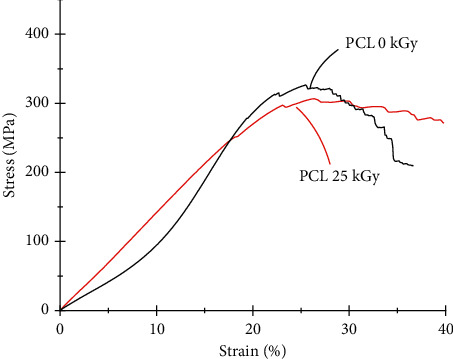
Stress-Strain behavior of sterilized groups.

Figure 8 shows the average tensile stress curves of PCL 25 kGy and PCL 0 kGy. The shape of the curve remained for the stress–strain behavior; when comparing PCL 25 kGy and PCL 0 kGy, they exhibited the same pattern. Mechanical behavior indicated a linear-elastic section up to ∼20%, which is followed by plastic deformation. The nonirradiated PCL had a longer toe at the beginning of the stress–strain curve and a slightly higher elastic modulus.

## 4. Conclusion

This study focused on analyzing the chemical and mechanical characterization of PCL microfilaments sterilized with gamma-rays. Results validated the use of 25 kGy-irradiated PCL microfilaments for biomechanical tissue-engineering applications. Dose distribution and dose uncertainty were important parameters to be studied because they ensured adequate sterilization of microfilaments samples during irradiation procedure. The mechanical behavior of microfilaments was not modified with the applied dose. Further studies may analyze the influence of diameter and composition variation of the microfilaments and the range of sterilization dosage.

## Figures and Tables

**Table 1:** Sample experimental conditions and conducted experiments.

Label	Condition	XRD	DSC	Microbial test	Tensile test
PCL 25 kGy	25 kGy gamma rays	✓	✓	✓	✓
PCL 0 kGy	No exposure	✓	✓	✓	✓

**Table 2:** Uncertainty budget for the irradiation of the microfilament samples.

Name	Uncertainty type	Probability distribution
Calibration (*σ*_cal_)	B	Normal
Reproducibility (*σ*_rep_)	A	
Machine (*σ*_mach_)	A	
Mapping (*σ*_map_)	A	

**Table 3:** Values of calculated or measured individual uncertainty.

Name		Distribution	Uncertainty^*∗*^%	
			A	B
Calibration	(*σ*_cal_)	Normal		2.80
Reproducibility	(*σ*_rep_)		1.39	
Machine	(*σ*_mach_)		0.53	
Mapping	(*σ*_map,max_)		1.81	
	(*σ*_map,min_)		0.31	

^*∗*^Uncertainty at a one standard deviation (k = 1).

**Table 4:** Limit values of the dose range for the monitoring dosimeter for routine dosimetry.

Name	Dose (kGy)
*D*_*target*,*lower*_	24.96
*D*_*target*,*upper*_	30.39

**Table 5:** Colony forming units of microorganisms detected on PCL 25 kGy samples microfilaments before and after sterilization with gamma rays.

Sample	Before *γ* sterilization			After *γ* sterilization		
	AMM	(A)/(AN)/(F)	(Y/F)	AMM	(A)/(AN)/(F)	(Y/F)
1	0	1 (A)	0	0	0	0
2	1	2 (A)	0	0	0	0
3	0	0	0	0	0	0
4	0	0	2	0	0	0
5	0	0	38	0	0	0
6	0	0	0	0	0	0
7	2	0	0	0	0	0
8	0	0	0	0	0	0
9	0	1 (F)	0	0	0	0
10	1	0	28	0	0	0

**Table 6:** Parameters obtained for different groups after tensile test.

Group	E	*σ*_y_	*σ*_ult_
	MPa	MPa	MPa
PCL 25 kGy	1716 ± 353	236 ± 68	298 ± 104
PCL 0 kGy	2337 ± 397	284 ± 36	325 ± 39

## Data Availability

The data that support the findings of this study are available upon reasonable request from the corresponding author.
